# Norm-SVR for the Enhancement of Single-Cell Metabolomic Stability in ToF-SIMS

**DOI:** 10.3390/metabo16010036

**Published:** 2025-12-30

**Authors:** Mingru Liu, Hongzhe Ma, Xiang Fang, Yanhua Chen, Zhaoying Wang, Xiaoxiao Ma

**Affiliations:** 1 Key Laboratory of Mass Spectrometry Imaging and Metabolomics , State Ethnic Affairs Commission, Center for Imaging and Systems Biology, College of Life and Environmental Sciences, Minzu University of China, Beijing 100081, China; 23302556@muc.edu.cn (M.L.); 23302569@muc.edu.cn (H.M.); 23011926@muc.edu.cn (X.F.); chenyanhua@muc.edu.cn (Y.C.); 2 Department of Precision Instrument, Tsinghua University, Beijing 100084, China; maxx@mail.tsinghua.edu.cn

**Keywords:** single-cell metabolomics, ToF-SIMS, data normalization, batch effect

## Abstract

**Purpose:** Data stability is a critical factor in Time-of-Flight Secondary Ion Mass Spectrometry (ToF-SIMS) single-cell analysis. However, various factors, such as sample processing, instrument condition, and data acquisition, can introduce uncertainties into ToF-SIMS data. Correcting this data is vital, yet current methods mainly focus on total ion intensity normalization or using consistent substrates. No specific correction method exists for ToF-SIMS single-cell metabolomics. **Methods:** This study utilizes the Normalized Support Vector Regression (Norm-SVR), commonly used methods for correcting large-scale metabolomics data, for the correction of ToF-SIMS single-cell metabolomic analysis and assesses its performance in comparison to traditional total ion intensity normalization. **Results and Conclusions:** The results suggest that Norm-SVR effectively diminishes batch effects and reduces variability, thereby underscoring the method’s efficacy and practicality. This approach is expected to improve data quality assurance in extensive ToF-SIMS analytical datasets.

## 1. Introduction

Single-cell analysis can reveal the molecular characteristics and functional properties of individual cells, providing crucial insights for studying cellular heterogeneity [1], cell-cell interactions [2], and disease response mechanisms [3]. Multiple analytical methods have emerged in this field, including flow cytometry [4,5], single-cell sequencing and multi-omics technologies [6], and single-cell imaging analysis. Single-cell imaging, with a particular emphasis on mass spectrometry imaging (MSI), establishes a connection between molecular characteristics and cellular functions by visually mapping the distribution and dynamics of molecules. In contrast to conventional imaging techniques that rely on probes or labels, mass spectrometry imaging utilizes a laser or ion beam to ionize biomolecules, which are subsequently identified by a mass spectrometer based on their mass-to-charge ratio. This technique, through scanning imaging of samples [7], maintains the cell’s natural state by eliminating the need for exogenous labeling, thereby preventing cytotoxicity and signal interference [8]. Furthermore, it facilitates the direct detection of endogenous molecules such as lipids and metabolites, enabling the precise capture of molecular alterations in both physiological and pathological processes, and thereby providing robust evidence for the elucidation of cellular functions [9].

Among the MSI techniques, Time-of-Flight Secondary Ion Mass Spectrometry (ToF-SIMS) is a widely utilized technique for the study of single cells, as it can simultaneously acquire comprehensive mass spectrometry data and corresponding two-dimensional molecular distribution images at the single-cell level [10]. Compared to other techniques, ToF-SIMS enables direct, label-free detection of endogenous metabolites, lipids, inorganic ions, and other molecules distributed on and within individual cells in situ. Furthermore, ToF-SIMS offers broad molecular coverage and rapid data acquisition, capable of detecting thousands of ion signals simultaneously. Leveraging these advantages, ToF-SIMS can reveal minute molecular changes in individual cells during metabolic pathways, drug responses, or pathological states. This makes it a crucial research tool for exploring disease mechanisms [11], drug action modes [12], and cellular functional states. However, current ToF-SIMS single-cell analysis methods still have certain limitations. The data collection process involves large volumes and complex analytical workflows [11]; every step of the experimental procedure—from sample preparation to mass spectrometry imaging acquisition, followed by data preprocessing and analysis—can significantly impact the accuracy and reproducibility of analytical results. For instance, cells cultured on the same silicon wafer may exhibit substantial variations in metabolomic data between those located in the central region and those at the periphery. Furthermore, when cells are cultured in different batches, the discrepancies observed across different silicon wafers may be even more pronounced. In addition, differences in ion yield can also lead to non-biological variations in inter-cell metabolic information, thereby reducing data reliability [13]. Therefore, performing effective calibration analysis on ToF-SIMS single-cell metabolomics data is a critical step in enhancing analytical stability and result comparability.

In large-scale untargeted metabolomics studies, several established correction methods have been proposed and successfully applied, primarily categorized into three types: correction based on quality control samples, correction based on internal standard compounds, and statistical correction [14]. In large-scale, multi-batch liquid chromatography-mass spectrometry (LC-MS)-based untargeted metabolomics studies involving diverse populations, these methods significantly enhance data comparability and stability by addressing systematic biases caused by signal drift, instrument variability, and batch effects [15]. For instance, the recently proposed Norm-ISW-SVR method combines internal standard compounds with a method of Support Vector Regression algorithm [16], demonstrating exceptional efficacy by maintaining high data reproducibility even at extremely low quality control (QC) frequencies. This strategy is not only applicable to traditional LC-MS platforms but also provides innovative insights for data correction in other mass spectrometry technologies.

Therefore, this study introduces a Normalized Support Vector Regression (Norm-SVR) corrected approach for single-cell metabolomics analysis in ToF-SIMS. By implementing Norm-SVR correction on single-cell metabolomics data across various acquisition regions and batches, we demonstrate that, in comparison to the traditional ToF-SIMS correction method utilizing total area/pixel normalization, Norm-SVR correction significantly mitigates batch effects and inter-cell variability. This enhances the reliability of ToF-SIMS for single-cell metabolomics and offers a dependable correction approach for future mass spectrometry imaging techniques in single-cell analysis.

## 2. Materials and Methods

### 2.1. Cell Culture

Prior to the experiment, polished silicon wafers (cut with a diamond saw to approximately 1 cm × 1 cm) were ultrasonically cleaned for 10 min each in methanol, acetone, and anhydrous ethanol, followed by two rinses in each solvent. The wafers were then air-dried at room temperature and stored in sealed test tubes for later use.

Human lung adenocarcinoma A549 cells were provided by the Cell Resource Center of the Institute of Basic Medical Sciences, Chinese Academy of Medical Sciences & Peking Union Medical College. A549 cells were cultured in a high-glucose DMEM (Gibco, Thermo Fisher Scientific, Waltham, MA, USA, Cat. No. C11995500BT). The medium was supplemented with 10% (*v*/*v*) fetal bovine serum (FBS, Sigma-Aldrich, Merck KGaA, Darmstadt, Germany, Cat. No. F8318; mycoplasma-free, endotoxin level ≤ 5 EU/mL, heat-inactivated at 56 °C for 30 min) and 1% (*v*/*v*) penicillin streptomycin (Gibco, Cat. No. 15140122; containing 10,000 U/mL penicillin G and 10,000 μg/mL streptomycin sulfate). The cells were maintained at 37 °C in an atmosphere containing 5% CO_2_ in the Constant-Temperature Incubator. When cell density reached approximately 80%, cells were washed twice with PBS, treated with 0.25% Trypsin-EDTA (Invitrogen, Thermo Fisher Scientific, Waltham, MA, USA, Cat. No. 25200072) for 2 min to detach, then washed with DMEM medium to terminate digestion. Cells were resuspended by pipetting to form a cell suspension. *A549* cells were seeded onto Si-coated culture dishes and allowed to adhere overnight for 12 h before undergoing fixation and drying.

### 2.2. Sample Preparation

The silicon wafer was removed from the culture medium and vertically immersed in Phosphate-Buffered Saline (PBS, Invitrogen, Cat. No. C10010500BT) for approximately 2 s. It was rinsed three times. Subsequently, the wafer was immersed vertically into 0.15 M ammonium formate (AF, Biorigin, Biorigin (Inc.), Beijing, China, Cat. No. BN24368) for approximately 30 s. It was then removed vertically and excess AF was blotted with absorbent paper. Under nitrogen atmosphere, the wafer was manually immersed into the cryogenic agent isopentane (pre-cooled with liquid nitrogen) for rapid freezing. It was removed steadily and transferred to a pre-cooled container under nitrogen atmosphere [17]. The sample-containing container was placed in a freeze-dryer overnight for 12 h at −55 °C and 0.01 mbar pressure. Subsequently, the sample was gradually heated until it reached room temperature to further volatilize residual isopentane solvent. The dried sample was immediately transferred to the ToF-SIMS for scanning.

### 2.3. ToF-SIMS Data Acquisition

ToF-SIMS measurements were implemented utilizing a ToF-SIMS V system (ION-TOF GmbH, Münster, Germany). The analysis of cellular samples was performed in an analysis-sputtering operational mode, where a 30 keV Bi_3_^+^ primary ion beam was applied for acquiring mass spectrometry images, and a 10 keV Ar_1700_^+^ sputtering beam was utilized to eradicate contamination and chemical damage induced by the Bi_3_^+^ beam on the sample surface. With an approximate spatial resolution of 300 nm, the Bi_3_^+^ ion beam current was maintained at 0.17 pA, and that of the Ar_1700_^+^ ion beam was set to 8.5 nA, with a cycle time of 100 μs. The sputtering region spanned 500 × 500 µm^2^, and the Bi_3_^+^ analysis beam was raster-scanned across a 350 × 350 µm^2^ central zone with a 256 × 256 pixels matrix. A non-interlaced scanning mode was adopted, configured with 2 analysis scans, 1 sputtering scan, and a 0.8 s interval pause per cycle. This cyclic process was repeated continuously until the full removal of cellular materials via sputtering. The resultant accumulated data were subsequently processed and visualized as a final integrated image. To mitigate charge buildup effects on the sample surface, a low-energy electron gun was operated throughout the measurement period.

### 2.4. Data Analysis

The mass spectra were processed using IONTOF SurfaceLab 7.0. The peak calibration was performed by C^−^, O^−^, OH^−^, C_2_H^−^, PO_3_^−^ and C_16_H_31_O_2_^−^ in negative ion mode, and C^+^, CH^+^, CH_3_^+^, C_2_H_3_^+^, C_3_H_5_^+^, C_5_H_12_N^+^ and C_5_H_15_PNO_4_^+^ in positive ion mode. We selected the mass spectrometry peaks with signal counts exceeding 1000 and a signal-to-noise ratio (SNR) greater than 3.0, within a mass range of 0–824 Da. The mass measurement range (0–824 Da) represents the instrument’s detectable range. Regions of interest (ROIs) were identified by applying a threshold based on the phospholipid fragment at *m*/*z* 184 in the positive ion mode and the phosphate group at *m*/*z* 79 in the negative ion mode. The relative signal intensity of individual cells was quantified by normalizing the total ion count across all pixels within each cell. Statistical processing and analysis of the data obtained from single-cell mass spectrometry were performed using R version 4.3.2.

### 2.5. Norm-SVR Correction Method

The Norm-SVR correction method employed in this study is a two-step approach—standardized preprocessing followed by support vector regression correction—specifically designed for the characteristics of ToF-SIMS single-cell metabolomics data. Its core objective is to eliminate non-biological systematic errors such as spatial location variations and batch effects while preserving the inherent metabolic heterogeneity of cells. This method does not require internal standards, instead relying on quality control (QC) samples to construct an error correction model. It is tailored to the characteristics of single-cell metabolomics: small sample size, high feature dimensionality, and sensitivity to technical errors.

#### 2.5.1. Standardization Preprocessing (Normalization): Z-Score Normalization

The Norm step employs Z-Score normalization (standard deviation normalization) to eliminate dimensional differences among feature ions, mitigate the over-influence of high-signal-intensity ions on the model, and preserve the relative distribution characteristics of ion signals. The calculation formula is as follows: $z_{i,j}=\frac{x_{i,j}-\mu_{j}}{\sigma_{j}}$.

Herein, $Z_{i,j}$ denotes the normalized signal value of the j-th feature ion in the i-th single-cell sample; $x_{i,j}$ represents the raw signal intensity of this ion; $\mu_{j}$ and $\sigma_{j}$ correspond to the mean and standard deviation of the j-th feature ion across all QC samples, respectively.

The advantage of selecting QC sample statistics for normalization lies in: mitigating interference from experimental group cell biological differences on normalization parameters, more accurately reflecting signal fluctuations caused by technical errors, and establishing a unified scale foundation for subsequent SVR correction.

#### 2.5.2. Support Vector Regression (SVR) Correction QC-Driven Systemic Error Modeling

The SVR stage employs the Support Vector Regression algorithm to construct a technical error prediction model using the repeatability signals from QC samples, thereby correcting systemic biases across all single-cell samples. Its core principle involves mapping high-dimensional feature ion data into a high-dimensional feature space via a kernel function to construct an optimal regression hyperplane. This hyperplane fits the nonlinear relationship between “signal drift” and “influencing factors,” enabling quantitative correction for systematic errors such as spatial position and instrument fluctuations. In this study, the SVR model configuration corresponds to the QC-driven SVR component within Norm-ISWSVR [6].

#### 2.5.3. QC Sample Selection Criteria

Considering the unique nature of ToF-SIMS single-cell analysis, QC samples were screened according to the “cell-scale quality control” principle, with specific criteria as follows: Using image analysis software, single cells of similar size were selected. QC samples were uniformly inserted at a 4:1 ratio (single-cell samples: QC samples) within the acquisition sequence across different collection regions. This ensures the model comprehensively captures systematic errors in both spatial and temporal dimensions.

Additionally, all data processing was performed using Python 3.10, R 4.3.2, and Anaconda 2023.09 (based on Python 3.11). For the dataset scale in this study, the processing time and resource consumption are as follows: Data preprocessing for Z-Score normalization: ~5–10 min; SVR model training and sample correction (with 5-fold cross-validation): ~12–15 min. No excessive resource consumption occurred throughout the process, making it compatible with the computing capacity of ordinary laptops.

## 3. Results

### 3.1. Establishment of a Norm-SVR Data Correction Method for ToF-SIMS Single-Cell Mass Spectrometry Data

**Figure 1:**
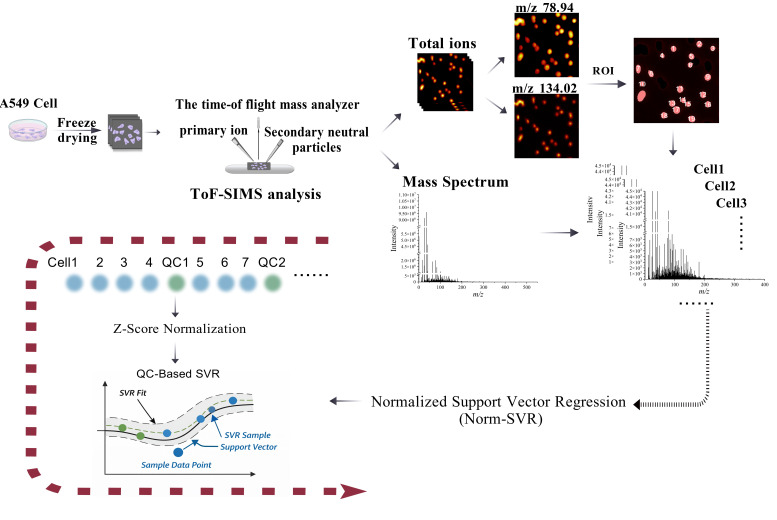
Schematic diagram of the data acquisition workflow for scanning Time-of-Flight Secondary Ion Mass Spectrometry (ToF-SIMS) and the extraction and processing of single-cell sample data. After preparing A549 cell samples, corresponding two-dimensional imaging spectra and mass spectra were obtained via ToF-SIMS analysis. Using phosphate ions (*m*/*z* 78.94) and adenine ion fragments (*m*/*z* 134.02) images as references, single cells were segmented as Regions of Interest (ROIs) on the total ion chromatogram. Data acquisition followed a top-down, left-to-right sequence, after which mass spectrometry information was extracted. The extracted single-cell data were subjected to Norm-SVR correction, which involved Z-Score normalization and quality control (QC)-based SVR.The black arrow in the picture indicates the sequence of the process.

Using our previously established single-cell sampling methodology for ToF-SIMS [17], we cultured *A549* cells to an appropriate density and prepared single-cell samples via freeze-drying. Following data acquisition using the ToF-SIMS ablation-analysis mode, we obtained ion images and mass spectrometry data. Subsequently, regions of interest (ROIs) and mass spectrometry data were extracted from individual cells within these ion images, as shown in Figure 1. Utilizing imaging spectra of phosphate ions (*m*/*z* 78.94) and adenine ion fragments (*m*/*z* 134.02) as reference markers [17,18], we defined cell dimensions and delineated cell boundaries. The ROIs for individual cells in the total ion image were sequentially analyzed from left to right and top to bottom. This sequence was employed to organize the single-cell mass spectral data, which was then extracted and compiled for further analysis. Preliminary examination of the total ion data indicated that even single cells originating from the same sample and silicon chip exhibited significant separation in principal component analysis (PCA) space, attributable to variations in collection areas. This observed spatial clustering did not stem from genuine biological differences but rather suggested that the collection location might introduce systematic bias, potentially obscuring biological variations among cells. Therefore, we employed the Norm-SVR method, a widely recognized technique for large-scale data correction in the domain of non-targeted metabolomics. Drawing upon prior research [19,20] that utilized “cell-sized blank regions of interest” in data analysis, we selected cells of similar size and consistent collection sequence intervals as QC samples throughout the analytical process. The ratio of single-cell samples to QC samples was maintained at approximately 4:1. This methodology facilitates the concurrent execution of background subtraction, quality calibration, and instrument monitoring within a single acquisition, obviating the need for internal standards or additional substrates. Subsequently, the Norm-SVR was used for correction. The Norm-SVR is divided into two parts. Firstly, Z-Score normalization is carried out, and then the data is corrected using Quality Control (QC)-based SVR. The specific principle is mentioned in the Section 2.

### 3.2. Norm-SVR Correction on Single-Cell Metabolomics Data Across Various Acquisition Regions

**Figure 2:**
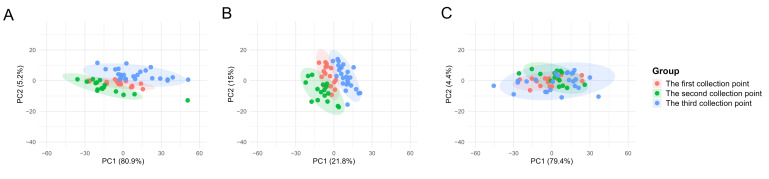
Principal Component Analysis (PCA) plots of single-cell mass spectrometry data from three sampling points on the same silicon wafer. PCA analysis was performed on Raw data, Total area/pixels normalized data, and Norm-SVR-corrected data. (**A**) The PCA plot of Raw single-cell data, where different sampling points exhibit distinct clustering. (**B**) The PCA plot of Total area/pixels normalized data, similarly arranged according to spatial sampling point distribution. (**C**) The spatially more uniform distribution of Norm-SVR-corrected data. The colors in the legend represent different sampling points and QC.

To assess the suitability of the commonly employed data correction method, Norm-SVR, in the context of ToF-SIMS single-cell mass spectrometry data analysis within the field of metabolomics, this study initially validated the approach using single-cell data obtained from three distinct collection points on a single silicon wafer sample, encompassing a total of 56 single cells. Figure S1 illustrates the spatial distribution of the three sampling points on the same silicon wafer, along with the corresponding ion imaging maps and single-cell region of interest (ROI) results. During the analysis process, based on the screening criteria (signal values exceeding 1000 counts and signal-to-noise ratio ≥ 3.0), we identified a total of 457 peaks, with peak values primarily distributed within the *m*/*z* 0–400 range. The peak areas of these 457 characteristic ion peaks identified in individual cells serve as the raw single-cell data. To evaluate the efficacy of Norm-SVR correction, the study utilized the widely adopted data processing technique of total ion intensity normalization as a benchmark for method performance. The PCA was conducted on Raw data, Total area/pixels normalized data, and Norm-SVR-corrected data, as depicted in Figure 2. The PCA results demonstrate clear distinctions: under both raw data conditions and Total Area/Pixels normalization, cell samples were grouped into three distinct clusters corresponding to their respective sampling locations. In the unprocessed Raw data, non-biological variation associated with sampling sites constituted 80.91% (Figure 2A), significantly surpassing the biological variation, which was 5.16%. This discrepancy obscured genuine metabolic differences among the cells. In contrast, the Total area/pixels normalized data showed that non-biological variation was comparable to biological variation, yet it remained spatially distributed according to the sampling locations. Following the Norm-SVR correction, cell samples from the three sampling sites were thoroughly intermingled across groups within the PCA space.

**Figure 3:**
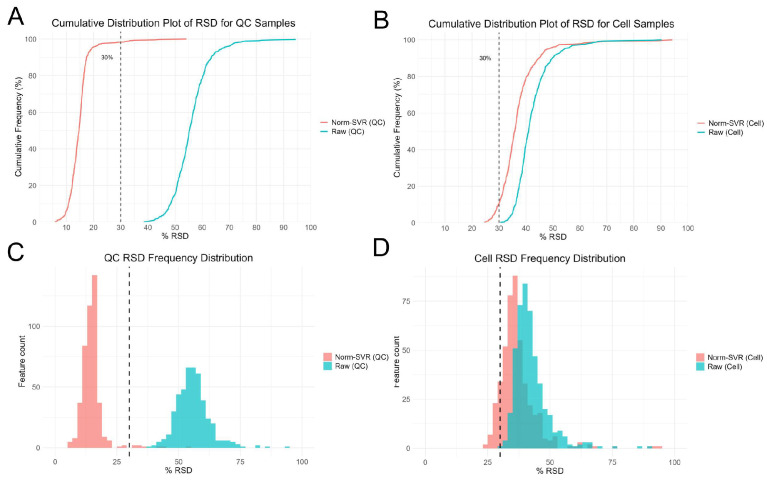
Residual standard deviation (RSD) analysis of single-cell feature ion data after Raw and SVR correction. Panels (**A**,**C**) show cumulative distribution plots and histograms of feature ions in QC samples, while panels (**B**,**D**) display cumulative distribution plots and histograms of RSD for cell samples.

To further evaluate the impact of SVR processing on ToF-SIMS single-cell metabolomics data, we performed Residual standard deviation (RSD) analysis on both Raw and Norm-SVR-corrected data (Figure 3). The results reveal that the RSD distribution of Raw data exhibits high dispersion in both QC and cell samples, indicating substantial inter-cell variability in uncorrected data. Specifically, the RSD values for most ion signals exceeded 50%, indicating significant non-biological variability in the data, potentially stemming from differences in sample collection locations. In contrast, after SVR correction, the RSD distribution of QC sample feature ions changed markedly: 86% of feature ions had RSD < 30%, demonstrating excellent Norm-SVR correction efficacy for QC samples and significantly reducing data variability. Similarly, compared to Raw data, the RSD distribution in the Norm-SVR corrected cell samples exhibited a flatter profile. This indicates more uniform RSD values in processed data, with a concentration of feature ions in the lower RSD range (approximately 0–30%). The frequency rapidly decreased as RSD values increased, revealing a distribution bias toward smaller RSD values. Overall, compared to the RSD variation in QC samples, single-cell samples exhibited smaller RSD fluctuations. However, the proportion of ions with RSD below 30% increased significantly, while those exceeding 50% decreased substantially. This may be attributed to Norm-SVR correction preserving intracellular biological variability. This demonstrates that Norm-SVR correction effectively eliminates systematic errors caused by sampling location differences. Through this approach, Norm-SVR homogenizes the RSD distribution across individual cells, enabling subsequent data comparisons and analyses to accurately reflect genuine biological differences between cells while eliminating non-biological variability stemming from sampling location differences. In summary, the Norm-SVR data correction method is applicable for processing ToF-SIMS single-cell data and effectively mitigates the impact of non-biological factors on measurement errors.

To further quantify the impact of various processing methods on inter-sample consistency, we computed the Pearson correlation coefficients for each sample pair using the Raw, Total Area/Pixels, and Norm-SVR datasets, subsequently visualizing these correlations as heatmaps. Additionally, we generated a scatter plot of the log_10_-transformed average intensities (comparing Raw data with Norm-SVR data) to assess linear consistency at the sample level. In the Raw dataset, the correlation structure was predominantly influenced by acquisition points, with samples collected from identical locations exhibiting high correlation, whereas correlations between samples from different acquisition points were significantly diminished, resulting in a distinct “blocky” pattern. This pattern corroborated previous PCA findings, indicating that non-biological variations were still predominant. Following normalization by Total Area/Pixels, there was a slight improvement in overall correlations; however, pronounced acquisition-dependent boundaries persisted, suggesting that systematic biases were only partially mitigated.

**Figure 4:**
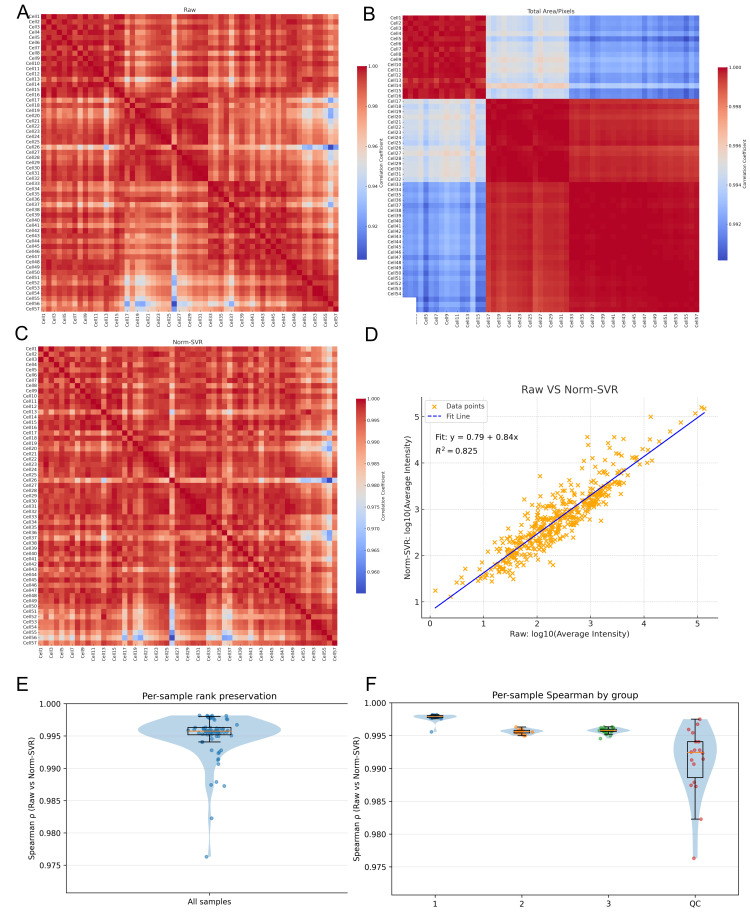
Comparison of sample correlations and signal consistency before and after normalization. (**A**–**C**) Pearson correlation heatmaps (sample × sample) of single-cell ToF-SIMS data under three processing modes: (**A**) Raw, (**B**) Total Area/Pixels, and (**C**) Norm-SVR. Samples are ordered by cell index, with higher uniformity (red color) indicating improved signal consistency among samples. (**D**) Linear relationships between log_10_ mean intensities of Raw versus Norm-SVR in cell samples. The strong correlation (R^2^ = 0.825) between Raw and Norm-SVR demonstrates that normalization effectively preserves true biological intensity relationships while reducing non-biological variability. (**E**) Per-sample rank preservation: Spearman correlation between Raw and Norm-SVR data across all samples, showing the preservation of rank structure after normalization. (**F**) Per-sample Spearman correlation by group: Spearman correlation between Raw and Norm-SVR data for samples grouped by experimental conditions (acquisition regions (1, 2, 3), and QC groups), indicating consistent normalization across different experimental groups.

Following the application of Norm-SVR correction, the overall correlation experienced a significant increase and exhibited a spatially heterogeneous distribution across acquisition points. The heatmap revealed a more uniform and elevated correlation distribution, with samples no longer clustering according to acquisition position. Furthermore, an analysis of the sample mean intensities for both Raw and Norm-SVR data revealed a stable linear relationship, with the majority of sample points aligning closely with the regression line. More importantly, Figure 4E,F show the Spearman correlation for each cell sample, with a Spearman correlation value greater than 0.995. This indicates that after correction by Norm-SVR, the ordering structure between the cell samples has hardly changed. This suggests that the correction method has removed non-biological noise while not erasing the true biological signals within the cells, allowing the corrected data to still accurately reflect the true differences between cells. This indicates that Norm-SVR effectively reduced non-biological variations, such as those related to acquisition location or batch, while largely preserving the relative intensity order among samples. As a result, this establishes a more dependable basis for future comparisons of biological differences. In summary, the correlation heatmaps and method consistency analysis validate the findings of the preceding PCA results. Notably, when contrasted with Total Area/Pixels, Norm-SVR exhibits a superior capacity to improve consistency across acquisition points. This method effectively reduces non-biological variations while maintaining the comparability of biological signals, thus providing a solid data foundation for subsequent differential ion screening and pathway interpretation.

### 3.3. Evaluation of Norm-SVR for Batch-Effect Correction

In large-scale ToF-SIMS single-cell experiments, systematic errors can easily arise due to batch differences, such as variations in sample preparation time, instrument fluctuations, and subtle temperature changes during freeze-drying. To address this issue, our study utilized A549 single-cell data from three distinct experimental batches, each separated by a 7-day interval to allow for natural fluctuations in instrument status. This approach was designed to assess the cross-batch correction capability of the Norm-SVR method. Each batch was prepared and data were collected using identical procedures, ensuring that all experimental conditions were consistent except for the batch itself. Across the three batches, we obtained a total of 156 single-cell samples and 45 QC samples, with each batch contributing 50–70 single-cell samples and 15 QC samples. In Figure S2 of the Supplementary Materials, we present ion imaging maps and single-cell ROI results at nine sampling points across three silicon wafers.

**Figure 5:**
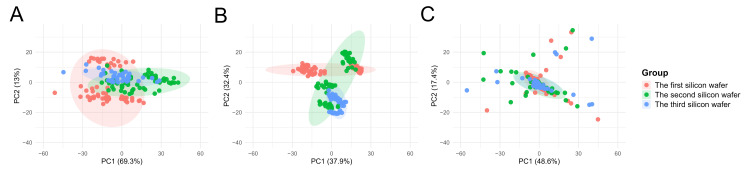
PCA score plots for single-cell ToF-SIMS data across different experimental batches before and after normalization. PCA was used to evaluate the influence of batch effects and the efficiency of different correction methods. Each color represents one batch, as indicated in the legend. (**A**) Raw data: Samples are clearly separated according to experimental batches, indicating strong batch effects. (**B**) Total area/pixels normalization: Partial reduction of variation is observed, but batch-dependent clustering remains. (**C**) Norm-SVR correction: Cells from different batches overlap extensively in PCA space, suggesting that Norm-SVR effectively mitigates inter-batch systematic variations and improves data comparability.

The results of the PCA indicated that, in the uncorrected Raw data, the cell samples from the three batches formed distinct clusters, with inter-batch distances significantly exceeding the intra-batch dispersion of cells. Following normalization using Total Area/Pixels, the degree of separation between batches was only marginally reduced, with the first principal component (PC1) still accounting for over 70.4% of the batch differences (Figure 5B). However, after correction using Norm-SVR, the cell samples from the three batches exhibited substantial overlap within the PCA space, resulting in the complete disappearance of batch boundaries (Figure 5C). This outcome indicates that the batch effect was effectively eliminated.

**Figure 6:**
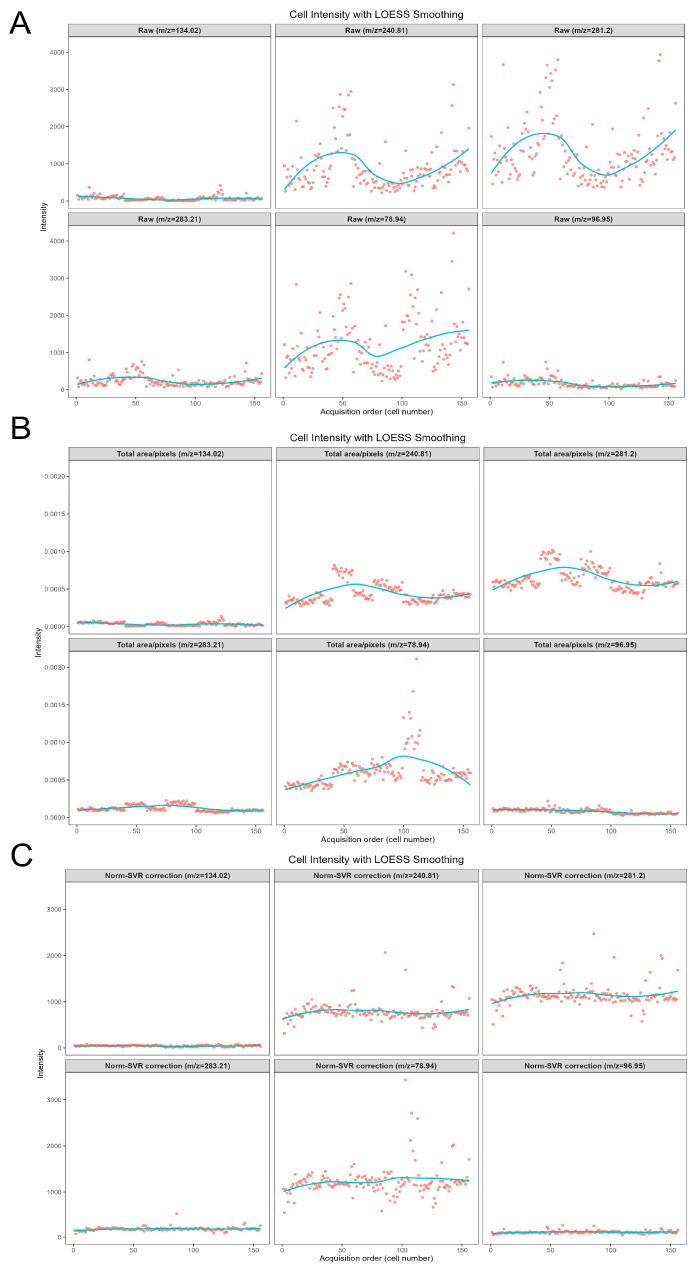
Scatter plots of six representative negative ion signal intensities within the *m*/*z* 50–400 range. Panels (**A**–**C**) show scatter plots for *m*/*z* 78.94, 96.94, 240.81, 281.20, and 283.21, under Raw, Total Area/Pixels normalization, and Norm-SVR normalization, respectively. Dots represent individual cell intensity values, and solid lines indicate Locally Estimated Scatterplot Smoothing (LOESS-smoothed) trend lines.

To further examine the correction scenarios associated with various characteristic ions of metabolites, Figure 6 concentrates on the analysis of five representative negative ions (*m*/*z* 78.944, 96.948, 240.814, 281.197, 283.214) within the negative ion mode. This figure systematically compares the impact of three data processing methods—namely Raw (original data), Total Area/Pixels, and Norm-SVR—on the stability of ion signal intensity. Concurrently, it investigates the correlation between the types of characteristic ions and the effectiveness of the correction.

Regarding signal stability, the Raw data exhibited significant deficiencies. The signal intensity of all target ions demonstrated irregular fluctuations. For instance, the signal intensity of *m*/*z* 78.944, presumed to be a characteristic ion of phosphate groups, was notably influenced by non-biological factors such as ion beam stability and sample surface charge accumulation. Additionally, the Raw signal distribution of *m*/*z* 281.197 (C_18_H_33_O_2_^−^, an ion characteristic of unsaturated fatty acids) was dispersed, with substantial signal intensity variations observed between adjacent cells. The Total Area/Pixels normalization process not only failed to enhance stability but also potentially increased the RSD of the target ions. The scatter trend line of *m*/*z* 240.814 (C_14_H_27_O_4_^−^, a lipid metabolite ion) exhibited a “zigzag” fluctuation pattern. Merely scaling the signal intensity by total ion count did not eliminate ion yield discrepancies and resulted in some distortion of ion signals. However, following Norm-SVR correction, the signal fluctuations of all target ions were effectively mitigated.

When examining the correlation between characteristic ion types and calibration effectiveness, significant corrections were observed for phosphate ions and other small-molecule inorganic ions (*m*/*z* 78.944, 96.948). This marked reduction is attributed to their heightened sensitivity to fluctuations in instrument parameters. Norm-SVR technology effectively compensates for real-time variations in instrument status by modeling systematic errors in quality control samples. In contrast, the RSD variation trend for lipid macromolecular ions (*m*/*z* 240.814, 281.197, 283.214) was more pronounced than that of small-molecule ions, significantly outperforming the Total Area/Pixels normalization methods. This discrepancy relates to the spatial distribution characteristics of lipid molecules, which are unevenly distributed across cell membranes and thus more susceptible to variations in ion beam sputtering efficiency. The nonlinear correction capability of Norm-SVR partially mitigates signal bias caused by spatial heterogeneity, whereas linear methods cannot effectively address this complexity.

Furthermore, we also investigated the correction effect of Norm-SVR on other metabolites. In the Supplementary Materials Figure S3, we added ions spanning different polarities and metabolic categories: small molecule polar metabolites (citric acid, *m*/*z* 115.02; aspartic acid, *m*/*z* 139.03), medium-polarity metabolites (glucose, *m*/*z* 179.03; sucrose, *m*/*z* 341.05), and non-polar metabolites (palmitic acid, *m*/*z* 255.23; phosphatidylserine fragment, *m*/*z* 313.23). The correction results were similar to those of phosphate ions, lipid ions, and other metabolites. The Norm-SVR correction method showed excellent performance in all cases. This indicates that this method has strong performance in the application of different metabolic categories.

As illustrated in Figure 5, the Norm-SVR method effectively transforms the sample structure across batches from a “batch aggregation” to an “overall mixture” configuration. This transformation is evidenced by the centralization and compactness of QC samples, indicating a suppression of non-biological fluctuations. Furthermore, Figure 6 highlights an enhancement in stability at the ionic level, particularly for small molecule inorganic and phosphate-based ions, which are more susceptible to variations in surface and instrument conditions. Additionally, lipid ions exhibit continuous improvement. These findings provide a robust foundation for subsequent analyses of characteristic ion changes within the metabolic profiles of large batches of single cells.

## 4. Discussion

While the Norm-SVR method demonstrates robust performance in correcting non-biological systematic biases (such as spatial location effects and batch effects) in ToF-SIMS single-cell metabolomics data, it is necessary to clarify its inherent limitations and optimization directions to provide an objective perspective for future applications.

The computational burden of Norm-SVR mainly stems from hyperparameter optimization (grid search combined with 5-fold cross-validation) during the model training phase. For the medium-scale dataset in this study (156 single-cell samples + 45 quality control (QC) samples), the total processing time is 17–25 min, which can be handled by ordinary laptops. However, when dealing with ultra-large-scale datasets with thousands of high-dimensional features, problems such as increased memory usage and prolonged computation time may arise. In addition, the technical error prediction model of this method is built based on the repeatability signals of QC samples, so the quality of QC samples directly affects the correction effect. If QC samples are contaminated, have abnormal metabolic profiles, or fail to meet the screening criteria set in this study (uniform cell size, signal count > 1000, signal-to-noise ratio ≥ 3.0, and a ratio of experimental samples to QC samples of 4:1), the model may misestimate technical errors, leading to deviations in correction results.

To address the above issues, potential future optimization directions include adopting data partitioning for parallel processing or moderate hardware upgrades (such as high-performance CPUs) for large-scale datasets. For ultra-large-scale scenarios, GPU acceleration can be integrated to speed up kernel function calculation and hyperparameter search, ensuring correction accuracy while improving scalability. In addition, it is recommended to pre-validate the quality of QC samples before model training and exclude low-quality samples; in the future, machine learning-driven automated QC screening modules can be developed to further reduce human errors.

Despite these limitations, Norm-SVR still has significant advantages: a standard-free design that eliminates the need for internal standards or specialized substrates, and the ability to retain true biological heterogeneity while reducing non-biological variability. These optimization directions will further enhance its practicality and reliability in large-scale, complex ToF-SIMS single-cell metabolomics analysis.

## 5. Conclusions

This study introduces and validates a Norm-SVR data correction method specifically designed for ToF-SIMS single-cell mass spectrometry data. The method addresses non-biological systematic biases, such as spatial location and batch effects, which can obscure genuine metabolic heterogeneity in single-cell samples. By employing phosphate and adenine fragment ions as spatial references and incorporating cell-sized quality control samples for multi-functional calibration, Norm-SVR demonstrated superior performance compared to Total Area/Pixels normalization across various validation experiments. The method effectively eliminated location-based clustering in PCA, reduced signal RSD for the majority of ions, concentrated signals within low-RSD ranges, and preserved intrinsic biological intensity correlations. In tests for batch effects, Norm-SVR successfully eradicated batch-dependent grouping and enhanced signal stability for both small-molecule inorganic ions and lipid metabolites. Overall, Norm-SVR offers a robust, standard-free approach to improve data comparability across different locations and batches while maintaining authentic biological variation, thereby establishing a critical foundation for reliable downstream single-cell metabolomics analysis through ToF-SIMS data.

## Data Availability

The data supporting this study are original experimental data from the first author’s Master’s thesis at Minzu University of China (MUC), currently not publicly archived. Temporary restricted access follows MUC’s regulations on postgraduate thesis data management and the need for subsequent extended research. The complete dataset, including raw data (e.g., mass spectrometry imaging files, sample pretreatment records) and processed data, is standardized and preserved by the corresponding author. After the first author completes thesis defense and complies with MUC’s data release rules, qualified researchers may request the data from the corresponding author, Zhaoying Wang (E-mail: zhaoying.wang@muc.edu.cn). Customized data processing codes are also available upon request to ensure research reproducibility.

## Supplementary Materials

The following supporting information can be downloaded at: https://www.mdpi.com/article/10.3390/metabo16010036/s1, Figure S1: Distribution of three sampling points on the same silicon wafer and ion imaging maps obtained via ToF-SIMS. Each row displays the ion imaging map and single-cell ROI results for the corresponding sampling point; the first three columns present ion imaging maps for Total ions, *m*/*z* 78.94 (PO₃⁻), and *m*/*z* 134.04 (adenine fragment) at the three sampling points, respectively, and the final column shows single-cell ROI results derived from the preceding ion images.; Figure S2: Ion imaging maps and corresponding single-cell ROI results from different batches. Each row of ion maps on the left shows imaging data from the same batch, with the corresponding single-cell ROI results (cell boundary delineation and ROI definition) displayed on the right.; Figure S3: Scatter plots of ion signal intensities for six representative ions within the *m*/*z* range of 50–400. Panels A, B, and C display the data for *m*/*z* 115.02 (citric acid), *m*/*z* 139.03 (aspartic acid), *m*/*z* 179.03 (glucose), *m*/*z* 255.23 (palmitic acid), *m*/*z* 313.23 (phosphatidylserine fragment), and *m*/*z* 341.05 (sucrose) under three processing conditions: Raw data, Total Area/Pixels normalization, and Norm-SVR normalization, respectively. Each dot represents the intensity of an individual cell, while the solid lines indicate the Locally Estimated Scatterplot Smoothing (LOESS) trend lines..
