# Supplementary material for: Norm-SVR for the Enhancement of Single-Cell Metabolomic Stability in ToF-SIMS
Source: Metabolites. 2025 Dec 30;16(1):36. doi: 10.3390/metabo16010036 (PMC12844169; doi:10.3390/metabo16010036)
Supplement: Supplementary file 1 [file metabolites-16-00036-s001.zip › metabolites-4056926-supplementary.pdf]

metabolites

Supplementary Materials

Norm-SVR for the Enhancement of Single-Cell Metabolomic Stability in ToF-SIMS

Mingru Liu, [1] Hongzhe Ma, [1] Xiang Fang, [1] Yanhua Chen, [1] Zhaoying Wang [1]* and Xiaoxiao Ma [2]

[1]Key Laboratory of Mass Spectrometry Imaging and Metabolomics (Minzu University of China), State Ethnic Affairs Commission, Center for Imaging and Systems Biology, College of Life and Environmental Sciences, Minzu University of China, Beijing 100081, P. R. China
[2]Department of Precision Instrument, Tsinghua University, Beijing,100084, China
* Correspondence: zhaoying.wang@muc.edu.cn

To evaluate the applicability of the commonly used data correction method Norm-SVR in ToF-SIMS-based single-cell mass spectrometry imaging analysis within the field of metabolomics, this study first validated the method using single-cell data obtained from three distinct sampling points on a single silicon wafer sample. Figure S1 displays the spatial distribution of the three sampling points on the same silicon wafer, along with the corresponding ion imaging maps and analysis results of regions of interest (ROIs) delineated based on the imaging maps.

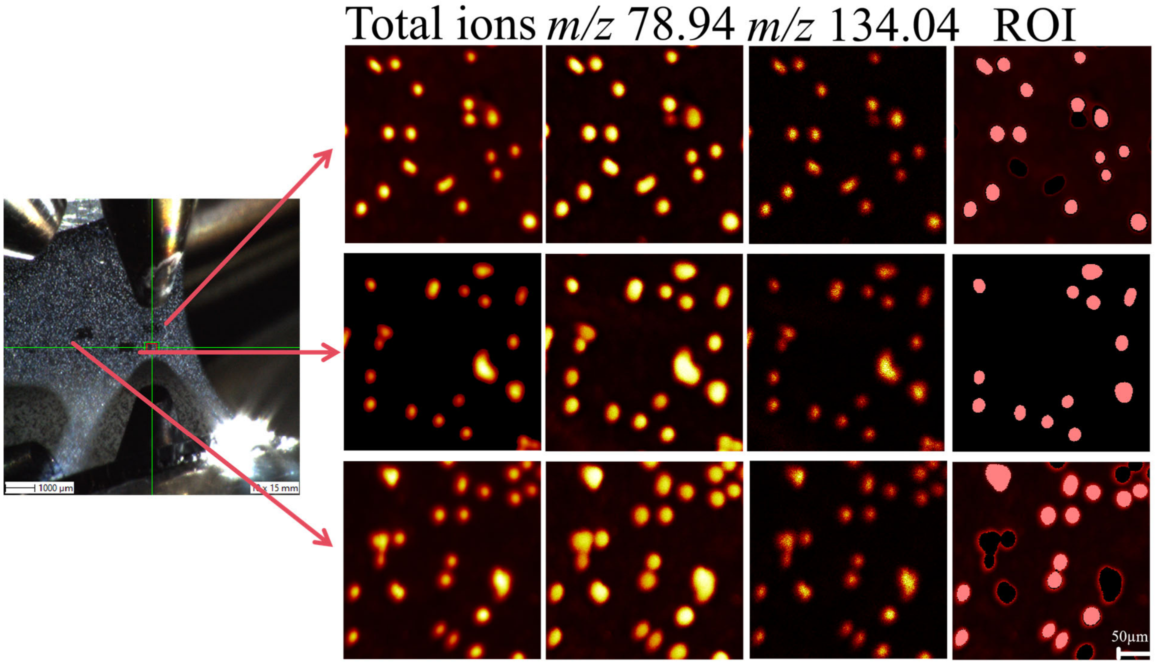

Figure S1. Distribution of three sampling points on the same silicon wafer and ion imaging maps obtained via ToF-SIMS. Each row displays the ion imaging map and single-cell ROI results for the corresponding sampling point. The first three columns present ion imaging maps for Total ions, *m*/*z* 78.94, and *m*/*z* 134.04 at the three sampling points, respectively. The final column shows single-cell ROI results derived from the preceding ion images.

The figure shows the distribution of three sampling points. Select the point closest to the center of the silicon wafer and two additional points at varying distances from the center for sampling. The sampling area measures 350 × 350 μm². The cell boundaries appear blurred in the total ion imaging spectrum. The phosphate ion PO_3^-, a known phospholipid

fragment, is abundantly present in the cell membrane. Therefore, cell size and boundaries were determined using the *m*/*z* 78.94 ion imaging spectrum, while cell count was determined via the adenine ion fragment *m*/*z* 134.04 imaging spectrum. Single cells were then selected for Region of Interest (ROI) analysis.

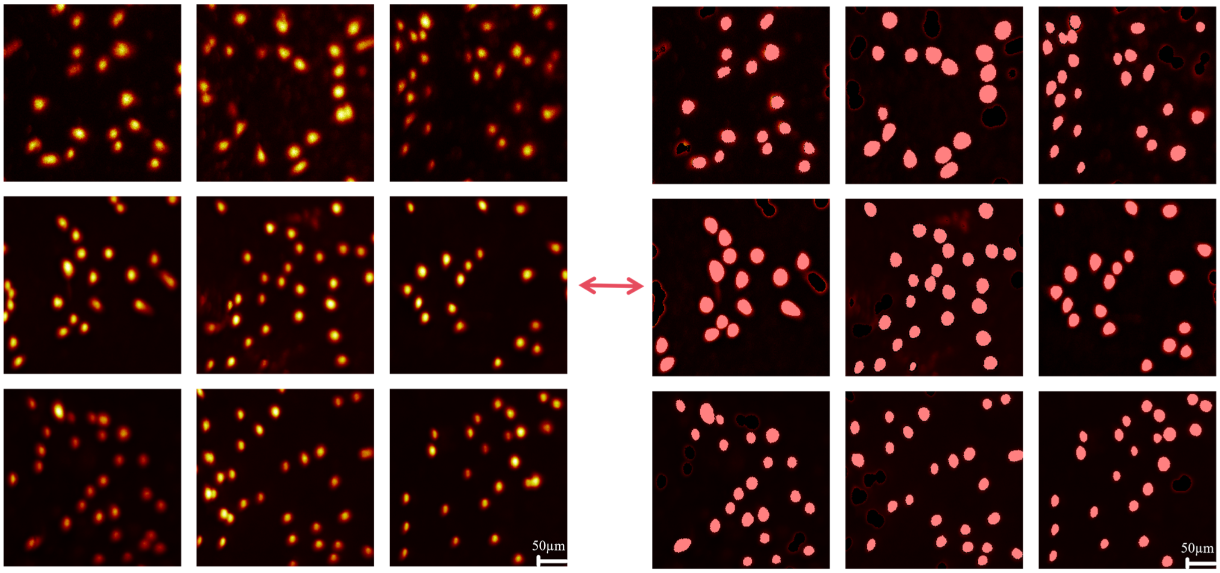

Figure S2. Ion imaging maps and corresponding single-cell ROI results from different batches. Each row of ion maps on the left shows imaging data from the same batch, with the corresponding single-cell ROI results displayed on the right.

Similarly, across different batches of single-cell sample collection results, cell size is determined in the same manner, cell boundaries are delineated, ROIs are defined for individual cells, and mass spectrometry data is extracted sequentially.

To study the correction effect of Norm-SVR on other metabolites. As shown in Figure S3, we analyzed ions covering different polarities and metabolic categories: small molecule polar metabolites (citric acid, *m*/*z* 115.02; aspartic acid, *m*/*z* 139.03), medium-polarity metabolites (glucose, *m*/*z* 179.03; sucrose, *m*/*z* 341.05), and non-polar metabolites (palmitic acid, *m*/*z* 255.23; phosphatidylserine fragment, *m*/*z* 313.23). The correction results were similar to those of phosphate ions, lipid ions, and other metabolites.

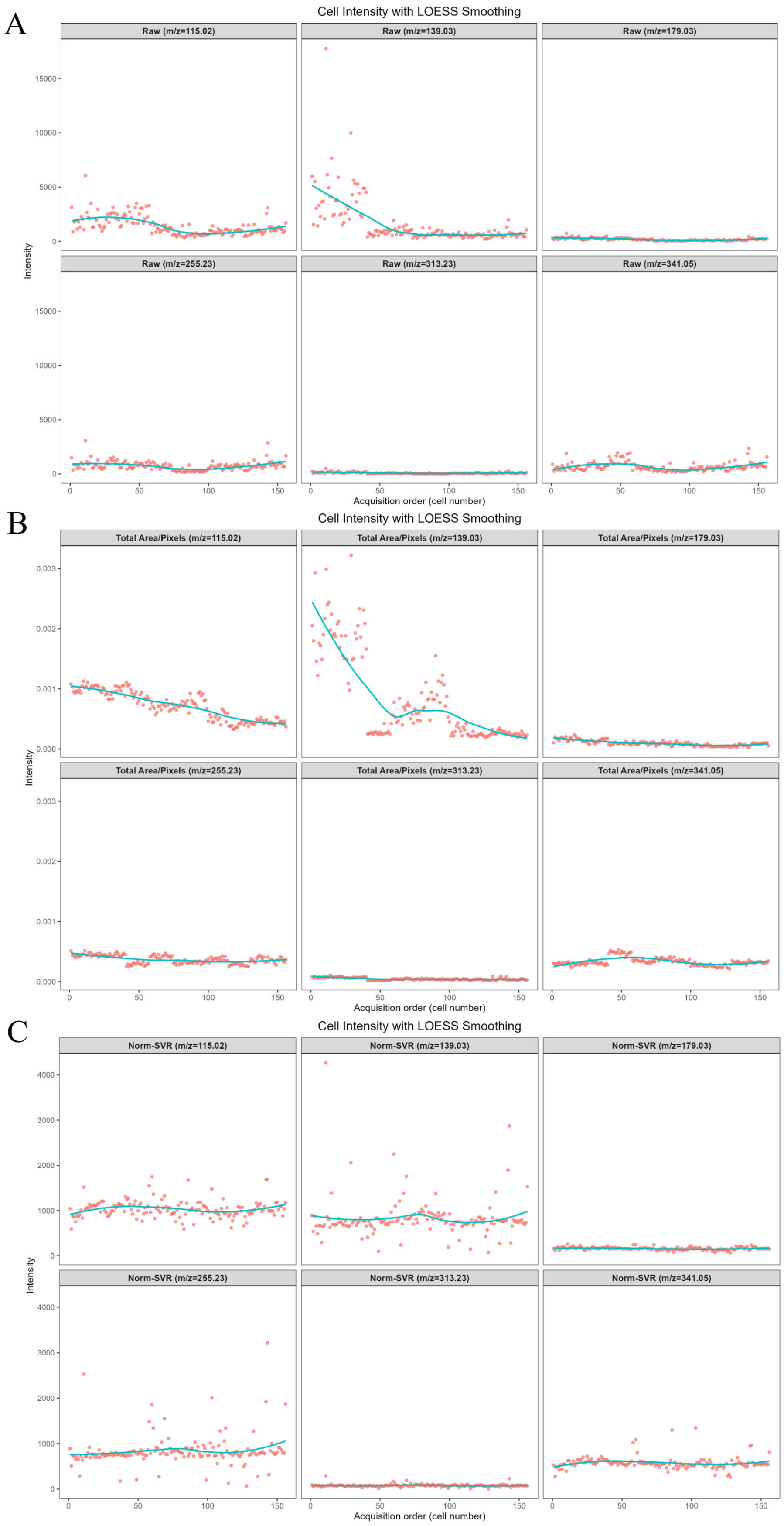

https://doi.org/10.3390/xxxxx

Figure S3. Scatter plots of ion signal intensities for six representative ions within the *m*/*z* range of 50–400. Panels A, B, and C display the data for *m*/*z* 115.02, *m*/*z* 139.03,*m*/*z* 179.03, *m*/*z* 255.23, *m*/*z* 313.23, and *m*/*z* 341.05 under three processing conditions: Raw data, Total Area/Pixels normalization, and Norm-SVR normalization, respectively. Each dot represents the intensity of an individual cell, while the solid lines indicate the Locally Estimated Scatterplot Smoothing (LOESS) trend lines, showing the overall intensity patterns across the acquisition sequence.

By analyzing these various ions, we found that the signal intensity of the original data showed significant irregular fluctuations. The signal intensities of all target ions (such as *m*/*z* 115.02, *m*/*z* 139.03, *m*/*z* 255.23, *m*/*z* 313.23, and *m*/*z* 341.05) exhibited obvious fluctuations. The signals of ions *m*/*z* 115.02 and *m*/*z* 139.03 showed a significant downward trend, and there was no regular change. The data of ions *m*/*z* 255.23 and *m*/*z* 313.23 presented a very scattered situation, and there were obvious outliers between some sampling points. Although the Total Area/Pixels normalization method reduced the fluctuations in the original data, it still had problems. This method adjusted the signal intensity through the ratio of total area/pixels, but this linear scaling method failed to fully address the differences in ion signal generation and instrument effects, resulting in the signals of some ions still showing irregular fluctuations. In the results of the Norm-SVR normalization processing, it can be seen that compared to the original data and Total Area/Pixels normalization, Norm-SVR significantly reduced the signal fluctuations: for *m*/*z* 115.02 and *m*/*z* 139.03, the signal intensity showed a clear smooth trend, and the irregular fluctuations were almost eliminated. The data for *m*/*z* 255.23 and *m*/*z* 313.23 have also become more stable, with the fluctuation range of the signals significantly reduced. Even for the *m*/*z* value of 341.05, the data processed by Norm-SVR exhibited extremely stable changes in signal intensity. This indicates that compared with traditional normalization methods, Norm-SVR not only performs better in terms of the smoothness of signal intensity, but also can handle signal deviations caused by non-biological factors.Through nonlinear correction, Norm-SVR precisely reduced the signal variations among different sampling points, making the data more consistent and enabling a better reflection of the true biological differences between cells.

Through this series of analyses, it can be seen that the Norm-SVR normalization method has significant advantages in correcting the ToF-SIMS single-cell metabolomics data, helping to eliminate non-biological variations and ensuring the high quality and reliability of the data.

https://doi.org/10.3390/xxxxx
